# Model of severe malaria in young mice suggests unique response of CD4 T cells

**DOI:** 10.1111/pim.12952

**Published:** 2022-10-04

**Authors:** Margaret R. Smith, Komi Gbedande, Corey M. Johnson, Logan A. Campbell, Robert S. Onjiko, Nadia D. Domingo, Michael M. Opata

**Affiliations:** ^1^ Department of Biology, College of Arts and Sciences Appalachian State University Boone North Carolina USA; ^2^ Division of Infectious Diseases, Department of Internal Medicine University of Texas Medical Branch Galveston Texas USA; ^3^ Present address: Cancer Biology Ph.D. Program Wake Forest College of Medicine Winston Salem North Carolina USA

**Keywords:** CD4^+^ T cells, immunity, malaria, *P. chabaudi*, young mice

## Abstract

Severe malaria occurs most in young children but is poorly understood due to the absence of a developmentally‐equivalent rodent model to study the pathogenesis of the disease. Though functional and quantitative deficiencies in innate response and a biased T helper 1 (Th1) response are reported in newborn pups, there is little information available about this intermediate stage of the adaptive immune system in murine neonates. To fill this gap in knowledge, we have developed a mouse model of severe malaria in young mice using 15‐day old mice (pups) infected with *Plasmodium chabaudi*. We observe similar parasite growth pattern in pups and adults, with a 60% mortality and a decrease in the growth rate of the surviving young mice. Using a battery of behavioral assays, we observed neurological symptoms in pups that do not occur in infected wildtype adults. CD4^+^ T cells were activated and differentiated to an effector T cell (Teff) phenotype in both adult and pups. However, there were relatively fewer and less terminally differentiated pup CD4^+^ Teff than adult Teff. Interestingly, despite less activation, the pup Teff expressed higher T‐bet than adults' cells. These data suggest that Th1 cells are functional in pups during *Plasmodium* infection but develop slowly.

**Abbreviations**
ACT: artemisinin combination therapy
IFN‐γ: interferon gamma
IL‐2: interleukin 2
IL‐10: interleukin 10
ITN: insecticide‐treated nets
Pups: young mice
PBMC: peripheral blood mononuclear cells
SHIRPA: SmithKline Beecham, Harwell, Imperial College, Royal London Hospital, phenotype assessment
TGFβ: transforming growth factor beta
TNF: tumor necrosis factor

## 1 INTRODUCTION

There was an estimated 241,000,000 malaria cases and 627,000 deaths in 2020, with children below the age of 5 years accounting for 80% of reported deaths.^1^ The disease becomes quickly severe in children, leading to high mortality.^2^ In addition, seizures, coma, and other signs of cerebral inflammation during infection predispose children to future disabilities, including poor cognitive development and epilepsy in those that survive *Plasmodium* infection.^3^, ^4^ While there have been significant advancements in our understanding of malaria pathogenesis, most of these studies have relied on adult animal models, with *Plasmodium chabaudi* being the best described and most physiologically relevant.^5^ Until now, there has not been a young rodent model for malaria infection. The absence of such a model has limited growth in knowledge on the disease progression and immune response due to an underdeveloped immune system early in life.

In establishing a model for malaria disease in younger mice, we consulted the literature on maturation of mice. A mathematical model has been widely used which relies on the developmental similarities between mice and humans to calculate analogies between mouse and human ages. In this formula, fusion of growth plates in the scapula is used as a marker of transition from adolescence to adulthood, when humans attain sexual maturity. This makes it possible to determine maturation status by dividing the average sexual maturity for humans in days, by that of mice. Based on this model, it is suggested that 2.60 mouse days are equivalent to one human year.^6^ Thus, the calculations provided by this model suggest that 14–15 mouse days is developmentally analogous to five human years, a common cutoff used in malaria susceptibility of children, as most cerebral malaria deaths from *P. falciparum* occur before that age. Establishment of such a model will enable the determination of immune development and response to *Plasmodium* infections in young mice. Such information can provide beneficial knowledge for improved design of malaria treatments and vaccines targeted for children.

Immunity to severe malaria develops gradually as children in highly endemic areas sustain multiple infections with *P. falciparum*. Clinical immunity reduces disease severity and likelihood death from the infection,^7^ while parasite immunity depends on the accumulation of antibodies specific to parasite strains a person is exposed to over time.^8^ Immunity to parasite growth requires both B and T cells, with CD4^+^ T cells central to regulation of both antibody responses and phagocytic killing of parasites. While Th1 responses are beneficial for parasite killing, they also are implicated in severe malaria.^9^ In some cases, this is accompanied by increases in FoxP3^+^ regulatory CD4^+^ T cells, though Tregs decrease after multiple incidences of malaria.^10^ These regulatory mechanisms create a balance between pathogen‐specific immunity and immune‐mediated pathology.^11^ These immune changes have been studied in *P. chabaudi* infection.^12^, ^13^ While *P. chabaudi* has been well‐established as a reliable model of pathogenesis and immunity, there should be more investigations of the role of age on immunity and pathogenesis.

Both young mice and children have a lower overall immune response to various stimuli including *Plasmodium* parasites.^14^, ^15^, ^16^ However, the mechanisms at play in determining increased severity to disease in young people are not clear, and it is not certain if this is a defect in their ability to develop immunity, or an increase in the propensity to cerebral malaria, which T cells contribute to.^17^ In this current study, we utilize C57BL/6 mice to describe the immune response to *P. chabaudi* infection in a young rodent model. Such a model would be beneficial in understanding the development of immunity to malaria in young children.

## 2 MATERIALS AND METHODS

### 2.1 Mice and parasites

C57BL/6 mice were maintained in a breeding facility at Appalachian State University Animal Facility Lab, or purchased from Jackson Laboratories (Bar Harbor, ME). Young mice were generated by breeding. Young mice were 14–15 days old of age, while adult mice were 8 weeks when infected with *P. chabaudi (AS)* for all experiments. Infected mice include both males and females and all experiments were carried out in accordance with the protocols approved by the UTMB Institutional Animal Care and Use Committee and The Appalachian State University Institutional Biosafety Committees. For survival studies, 1 × 10^6^ infected red blood cells (iRBCs) were used while 1 × 10^5^ iRBCs were used for CD4 T cell immune response studies.

### 2.2 SHIRPA

SmithKline Beecham, Harwell, Imperial College, Royal London Hospital, phenotype assessment (SHIRPA) was conducted to characterize the behavioral changes in pups during three stages with a series of individual tests that provide quantitative data about the pup's individual performance.^18^, ^19^ SHIRPA mimics the general and neurological examination in humans, and measures Reflexes and Sensory Function, Neuropsychiatric Function, Muscle and Lower Motor Neuron Function, Spinocerebellar function, Autonomic Function, as well as Muscle Tone and Strength. When a pup showed a poor performance in a locomotor test early in infection this reflected a muscular weakness due to a dysfunction in the central nervous system, not muscular wasting seen later. The tests did not change the course of infection, and we allowed them 30 min rest before another test.

### 2.3 SHIRPA tests to assess strength and sickness

The mouse is transferred quickly into a new environment with little human contact in order to observe the immediate reaction. For transfer arousal, a score of 5 showed that the mouse did not freeze when in a new environment while a lower score indicates a longer pause before moving. For tail and pelvic elevations, the mouse is observed during forward motion, as it explores its new environment. A score of 2 indicates that the tail is elevated, a 1 indicates that it is extended horizontally, and a score of 0 shows that the tail is being dragged. If the pelvic region was more than 3 mm in elevation the score of 3 was assigned, 2 represented a normal elevation of 3 mm, 1 showed that the pelvic elevation was barely touching the floor and 0 was the pelvis was flattened on the ground. Mice were in the beaker for 5 min before body position was recorded. A score of 4 indicated that the mouse was standing on their hind legs, a score of 3 meant that the mouse was sitting, a score of 2 meant that the mouse was lying prone, and a score of 1 indicates that the mouse was lying on its side. When the mouse is placed in the beaker, it is observed for spontaneous activity. A score of 3 shows that the mouse had a rapid and darting movement, a score of 2 meant vigorous grooming and moderate movement, 1 represents casual grooming or slow movement. When a mouse was resting or showed no movement it was given a score of 0. As the mouse was exploring the new environment the pelvic elevation was observed.

### 2.4 SHIRPA tests to assess neurological deficits

The mouse was placed in an open arena and allowed to explore the new environment. The arena had equal length and width in squares. For locomotor activity, the number of squares all four paws entered was counted over 30 min. For tremor, the mouse was observed when placed in a glass beaker. A score of 2 shows no tremor, a 1 represents a mild tremor, and a 0 shows that an important tremor was observed. For negative geotaxis, a mouse was placed on a horizontal cage top and when the mouse moved in one direction the top was raised vertically so that the animal was facing downwards. A stopwatch was set for 30 s, and the mouse was observed. A score of 4 indicates that the mouse turned around and climbed up the grid, a 3 represents a mouse that turned around but froze. When a mouse moved but did not turn around it was given a score of 2, 1 was given when the mouse did not move for 30 s, and 0 represents when the mouse fell off grid. For touch escape, a mouse was stroked by a finger while exploring its new environment. If the mouse vigorously escapes the finger stroke, it was given a score of 3, if the mouse had a rapid response to a light stroke it was given a score of 2, and a score of 1 was given to mice that did not escape upon a light finger stroke. For visual placing, the mouse is held by its tail and lowered to the cage top, and the extension of the forelimbs by the animal was observed. A score of 4 showed that the mouse had early vigorous extension around 25 mm above the cage top, 3 represents an extension of forelimbs before vibrissa contact around 18 mm, 2 shows that the extension of forelimbs occurred upon vibrissa contact, 1 means it occurred upon nose contact, and 0 shows that there was no response.

### 2.5 Monitoring weight

Adult and young mice were infected with 10^6^ *P. chabaudi* parasites for survival and behavioral studies. Mice were weighed daily on a balance in a glass beaker (1 L). Percent weight change was calculated using the original weight before infection on day 0 of the experiment [(Weight today – weight d0)/Weight d0] × 100%.

### 2.6 Determining parasitemia

Parasite burden was determined using thin blood smears obtained by bleeding the tail of the mice between days 5–21 post‐infection with *P. chabaudi*. The slides were stained with Diff‐Quick (Fisher Scientific, GA) and parasites were counted by microscopy in 10 to 50 different fields depending on the parasite load and day of infection. To determine percent parasitemia, the number of infected red blood cells was divided by the total number of red blood cells in all counted fields. The outcome was multiplied by 100 as shown in the formula below.

$$\%\text{Parasitaemia}=\frac{\text{iRBC}}{\text{Total}\;\mathrm{RBC}}\;X\;100$$

### 2.7 Intracellular cytokine staining and flow cytometry

Single‐cell suspensions were obtained from the spleens in complete Iscove's media (5% FCS, and 3 mM HEPES). Cell pellet was resuspended and incubated in red blood cell lysis buffer to eliminate red blood cells. Cells were then washed in phosphate‐buffered saline supplemented with 2% fetal bovine serum, and 0.01% sodium azide, followed by enumeration (5 × 10^6^ cells), and stimulation with 1X of cell stimulation cocktail (Tonbo Biosciences, San Diego CA) for 5 h. The cells were incubated for 5 h at 37°C, and 5% CO_2_ in the presence of GolgiPlug (BD Biosciences) for intracellular cytokine staining. Upon harvest, cells were surface stained with anti‐CD4 eflour 450 (clone RM4‐5, Invitrogen), anti‐CD25 PE/Cy7 (clone, PC61.5, eBiosciences), anti‐CD44 APC‐efluor 780 (clone‐IM7, eBiosciences), anti‐CD127‐PE/Cy5 (clone A7R34, Biolegend), anti‐CD62L (clone MEL‐14), anti‐CD27‐APC (clone LG‐7F9, eBiosciences), and incubated for 30 min. Samples were washed with FACS buffer (PBS with 2% FBS and 0.01% Sodium Azide), and fixed by adding 2% PFA in PBS followed by a 30 min incubation. The cells were then incubated in 1X Perm/Wash buffer (BD Biosciences) for 30 min to permeabilize the cell membrane. After permeabilization, cells were incubated with anti‐CD16/32 Fcblock (BioXcell, Lebanon, NH) for 20 min, followed by addition of anti‐IFNγ FITC (clone MG1.2, Invitrogen), IL‐2‐PE (JES6‐5H4, eBiosciences), IL‐10‐BV650 (clone JES 5‐16E3, BD Biosciences), TNFα‐PE/Cy7 (clone MP6‐XT22, Biolegend), for cytokine intracellular staining and T‐bet‐PerCp‐Cy5.5 (clone 4B10, BD Biosciences), or FoxP3‐FITC (clone FJK‐16 s, eBiosciences) using transcription factor staining buffer for transcription factor staining. Then cells were incubated for a further 40 min in the refrigerator. Cells were then washed three times with perm/wash buffer, and samples were analyzed by the Fortessa, Attune NxT, or FC500 flow cytometer. Data were analyzed using FlowJo (BD/TreeStar, Ashland, OR).

### 2.8 Statistical analysis

Data collected after FlowJo analysis was quantified in Microsoft excel and statistical analyses performed using GraphPad Prism (TreeStar, Where) version 9.0. Statistics was determined based on standard error of the mean (SEM). *p* values were calculated using one‐way ANOVA followed for Tukey post‐test where necessary due to non‐normality of the data. In some experiments, unpaired Student's *t*‐test was used and *p* ≤ 0.05 was considered significant.

## 3 RESULTS

### 3.1 Young mice infected with *P. chabaudi* have decreased weight gain

**FIGURE 1:**
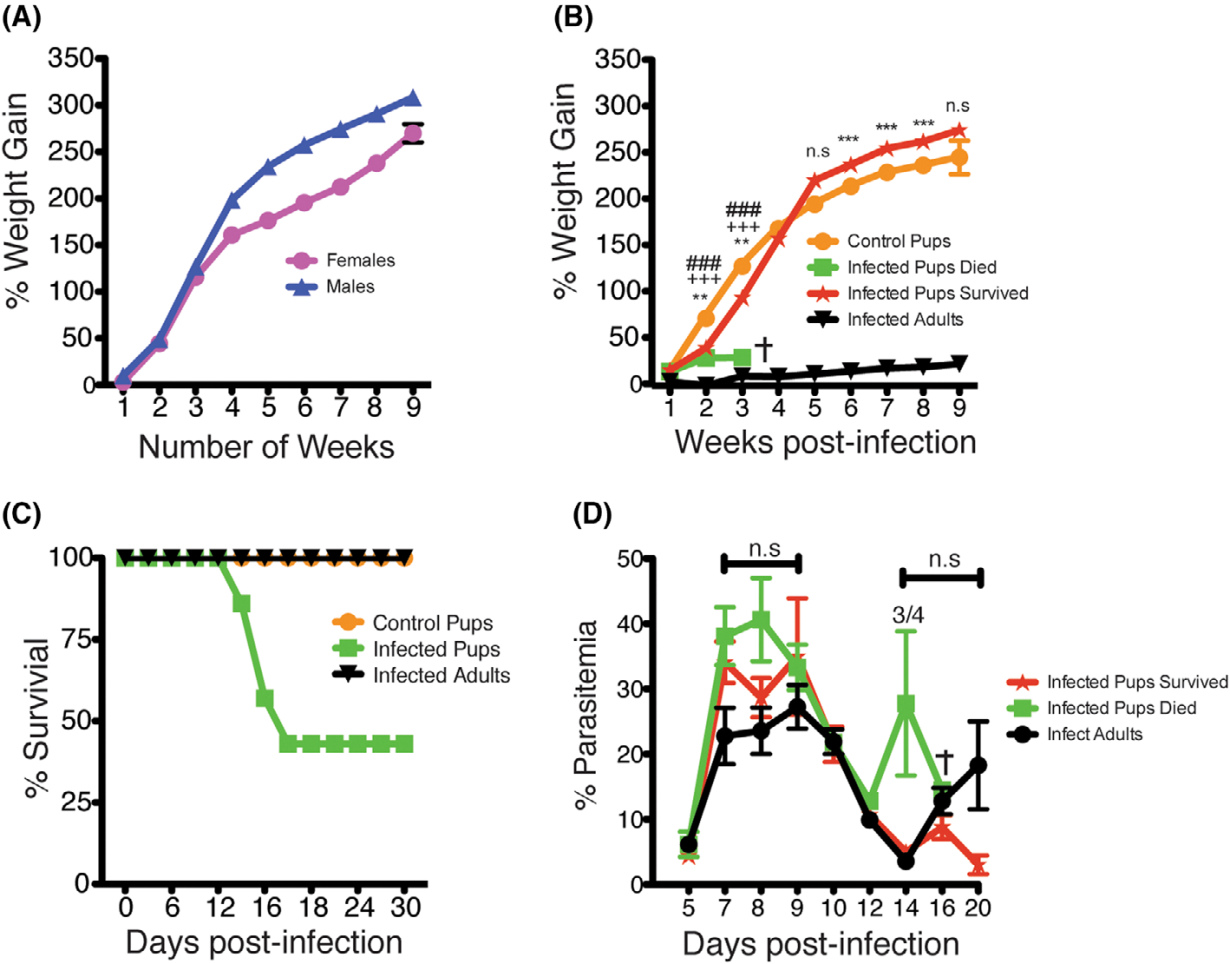
Pups infected with *Plasmodium chabaudi* show a stunted growth and a higher chance of death. (A) Healthy C57BI/6 pups were weighed from day 10 to day 70 post birth to determine weight gain between female and male mice. (B) Pups were infected and weights monitored over 9 weeks, data shows the difference in weight of control pups, infected pups that died, infected pups that survived and infected adults. (C) Percent of survival of control pups, adults infected with *Plasmodium chabaudi* and infected pups. (D) Percent parasitemia between infected pups that survived or died and infected adult mice from days 5 to 20 post‐infection. The data represent three independent experiments with 5–7 pups infected, 3 pups for control, and 3 infected adults. The error bars represented the standard error of the mean (SEM). ***p > 0.0001, *p* < 0.0022 comparing infected pups that survived to control pups, +++*p* < 0.0030 comparing infected pups that died to infected pups that survived, ###*p* < 0.0002 comparing control pups to infected pups that died using One‐Way ANOVA followed by Tukey post‐test. Fraction shows number of mice that died out of total mice

Children under the age of five can suffer severe malarial disease and are more susceptible to death because of *P. falciparum* infection. It has also been proposed that sickness from malaria reduces the growth rate of children, though this is challenging to prove definitively due to the many variables involved with children in endemic areas.^20^ Therefore, we set out to determine the effect of *Plasmodium* infection on the growth of young mice. To establish the normal growth of pups, we studied the progression of growth in young uninfected mice starting from day 10 after birth for 10 weeks. Male and female pups are shown separately but have similar growth patterns (Figure 1A). After 3 weeks (21 days), when we weaned the pups from their mothers, male mice briefly gained weight faster compared to their female littermates. Next, we infected pups at 15 days of age, and adult mice (8 weeks old). Based on a mathematical model by Dutta & Sengupta that determines adulthood and maturity, we approximated that 15‐day old pups would developmentally be equivalent to 5.8 human years.^6^ After infecting the 15‐day old pups, we monitored change in their weight for 10 weeks. We observed a significant slowing in weight gain in the infected pups between days 9–21 post‐infection (p.i), compared to uninfected controls (Figure 1B).

Pups that died strikingly had not gained more than 30% weight by the third week, and pups that lived also had significantly reduced growth. This result is consistent with stunted growth observed in children in malaria endemic areas,^20^ and suggests that *Plasmodium* infection directly contributes to stunting. Infected adult mice lost weight at the peak of infection, but regained their normal weight after recovery by third week p.i. Some pups succumbed to the infection, resulting in 60% mortality (Figure 1C), and these mice failed to gain weight by the third week of infection. Parasitemia trends showed an increase in pups, but this did not reach significance when compared to parasitemia in adult mice (Figure 1D). Pups that died also had an additional recrudescence at day 14 p.ii. These data suggest that malaria infection leads to reduced growth rate of pups during infection.

**FIGURE 2:**
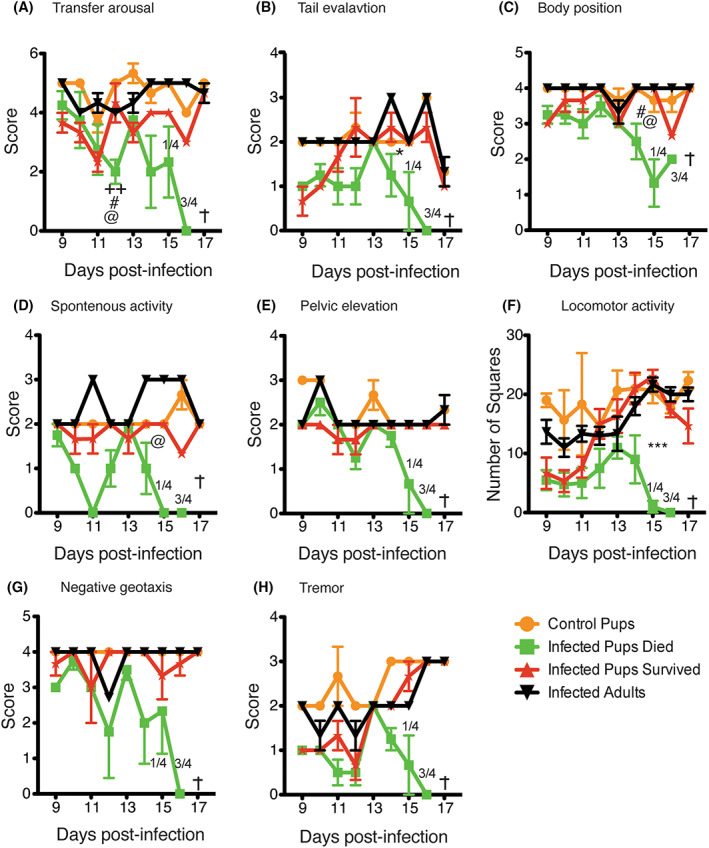
Malaria infected pups have decreased behavioral scores compared to the controls and infected adults. SHIRPA was conducted on the mice as described in the materials and methods section after *Plasmodium chabaudi* infection to understand the natural behavior of infected pups in comparison to adults. Graphs show (A) Transfer arousal to determine the reaction of mice upon quick transfer to a new environment with little human contact. (B) Tail elevation (C) body position (D) spontaneous activity, and (E) pelvic elevation as the mouse moves around exploring new environment. (F) Locomotor activity when a mouse is placed in an arena and allowed to explore the new environment. (G) Negative geotaxis, when a mouse moves down an inclination, and (H) Tremor when observed in a glass jar. These data represent two independent experiments with seven infected pups, three control pups, and three infected adults. The error bars represent the standard errors of the mean (SEM). Fraction indicate number of dead mice on the indicated day and + indicate all mice dead on that day. Groups were compared using One‐Way ANOVA followed by Tukey post‐test with *p > 0.05 considered significant. * compares infected pups that survived to control pups, + compares infected pups that died to infected pups that survived, # comparing control pups to infected pups that died, and @ compares infected adults to infected pups that died. Fraction shows number of mice that died out of total mice

Malaria can lead to neurological complications and behavior problems in children.^21^, ^22^, ^23^ Cerebral malaria can be detected in mice as behavioral changes.^19^, ^24^ Therefore, we sought to determine the effects of the infection on the behavior of pups compared to adult mice. Pups (15 days old) or adult mice were infected with a dose of 1 × 10^6^ *P. chabaudi* infected red blood cells (iRBCs) that is 60% lethal to pups, and we performed an animal health and behavior assessment test starting from day 9 p.i. The SHIRPA, a rigorous and semi‐quantitative battery of forty behavioral tests, has revealed significant deficits in animals infected with both *P. chabaudi* ^19^ and *P. berghei* ANKA‐induced experimental cerebral malaria.^25^ Many scores deteriorated starting near the time of death as shown in Figure 2. Infected pups that died suffered a more severe course of disease which can be measured by significantly decreased scores on day 11 or d14 in several behavioral tests including transfer arousal, tail elevation, body position, spontaneous activity, and pelvic elevation. All of these tests are scored when a mouse is placed in a glass jar with minimal human disturbance. The mice that survived the infection trends toward lower scores in some behavioral parameters.

Transfer arousal is a measure of neuropsychiatric state indicative of responsiveness to a new environment (Figure 2A) and infected pups were much less responsive than uninfected pups both at day 9 and by day 15 with less dramatic changes in adults. Tail elevation (Figure 2B) and body position (Figure 2C) are measured without human interference as indicators of motor behavioral state. Tail elevation is relatively depressed in infected pups compared to uninfected pups, but not adults at day 9. Both tail elevation and body position scores are dramatically decreased in the pups that die compared to all other groups starting on day 14. Spontaneous activity is a measure of neuropsychiatric state that is significantly decreased only in the pups that are going to die on days 11 and starting on day 14 p.i. (Figure 2D). Pelvic elevation and locomotor activity are measures of motor behavior that could be affected by muscle wasting from sickness (Figure 2E,F). Negative geotaxis (Figure 2G) and tremor (Figure 2H) are behavioral measures that are specific to cerebral malaria, and not to generalized sickness behavior. Some of these changes have been previously observed in *P. berghei* ANKA or IL‐10 deficient mice with *P. chabaudi* infection. These data suggest that by the second week of infection, pups show weakened muscular strength and locomotor activity indicators of sickness behavior.

### 3.2 Effector CD4 ^+^ T cells from pups infected with malaria are activated but produce less protective IFNγ and TNF double cytokines

**FIGURE 3:**
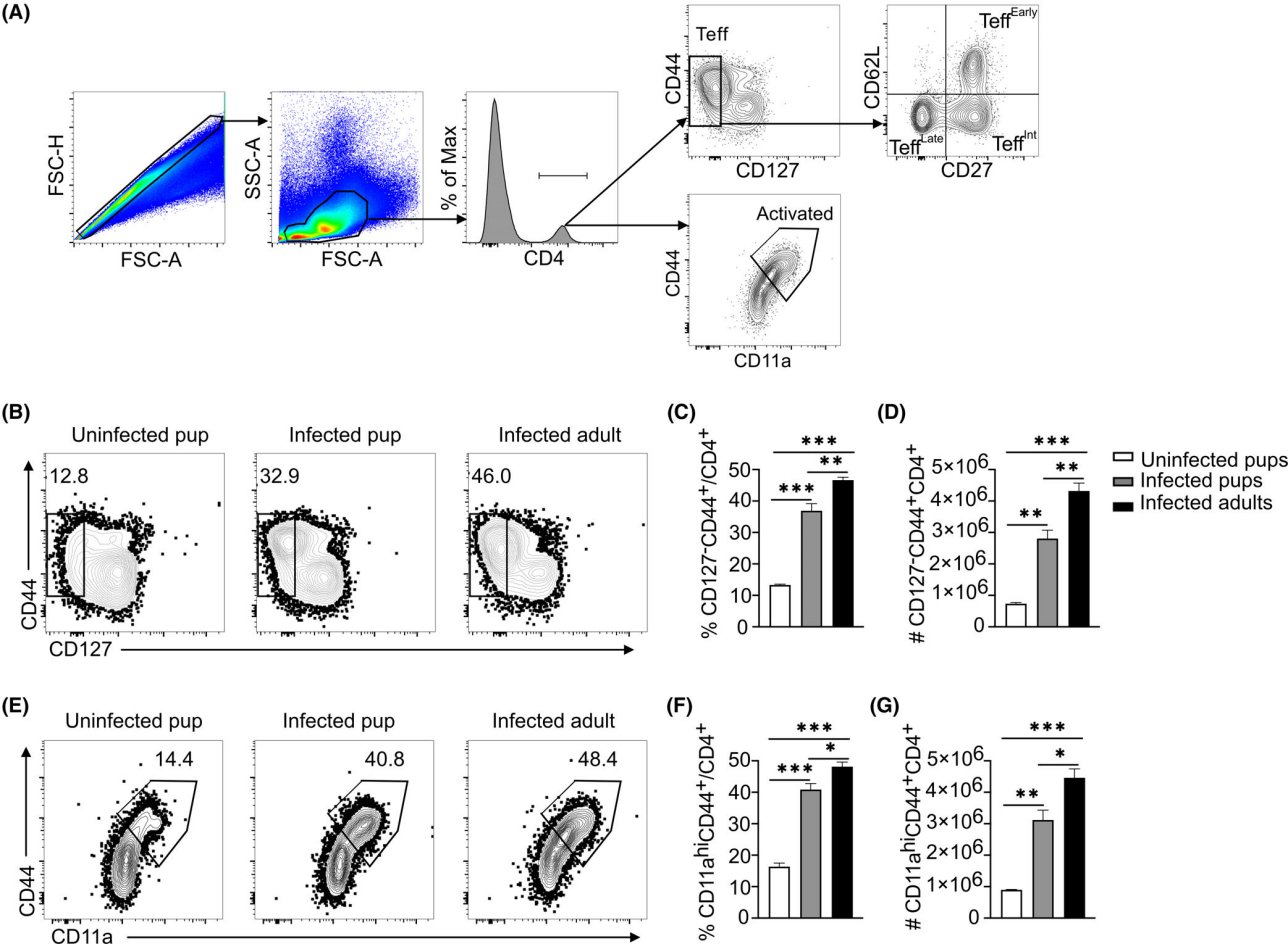
Pup CD4^+^ T cells are activated at a slower rate than adult T cells in response *P. chabaudi* infection. Pups and adult C57BL/6J mice were infected with *P. chabaudi* (10^5^ iRBCs), and CD4^+^ T cells from the spleens were analyzed at day14 post‐infection. Uninfected age‐matched pups were used as control. (A) Plots indicating the gating strategy. (B) Plots and (C) graphs showing percentage and (D) number of Teff (CD127^−^CD44^+^CD4^+^). (E) Plots and (F) Graphs showing percentage and (G) number of activated T cells (CD11a^hi^CD44^+^CD4^+^). Data represent two independent experiments with 3–4 mice per group. The error bars represent standard error of the mean (SEM). One way ANOVA following Tukey's multiple comparisons test were used to compare between groups, *p < 0.05, **p < 0.01, ***p < 0.001.

Previous studies have suggested that children could be more susceptible to different infections due to the early stage of development of their adaptive immunity.^14^ To test for differences in CD4^+^ T cell activation and function in response to infection between adult and young mice, we infected 8‐weeks‐old adult or 14‐day‐old pups with 10^6^ *P. chabaudi* iRBCs, and determined effector T cell activation and numbers on day 8 and 14 p.i. using the gating strategy as shown in Figure 3A. Both pup and adult CD4^+^ T cells were activated as shown by the downregulation of CD127 (IL‐7R‐) (Figure 3B–D) and upregulation of CD44 and CD11a (Figure 3E–G), even though the adult cells were significantly higher compared to the infected pup cells (Figure 3).

**FIGURE 4:**
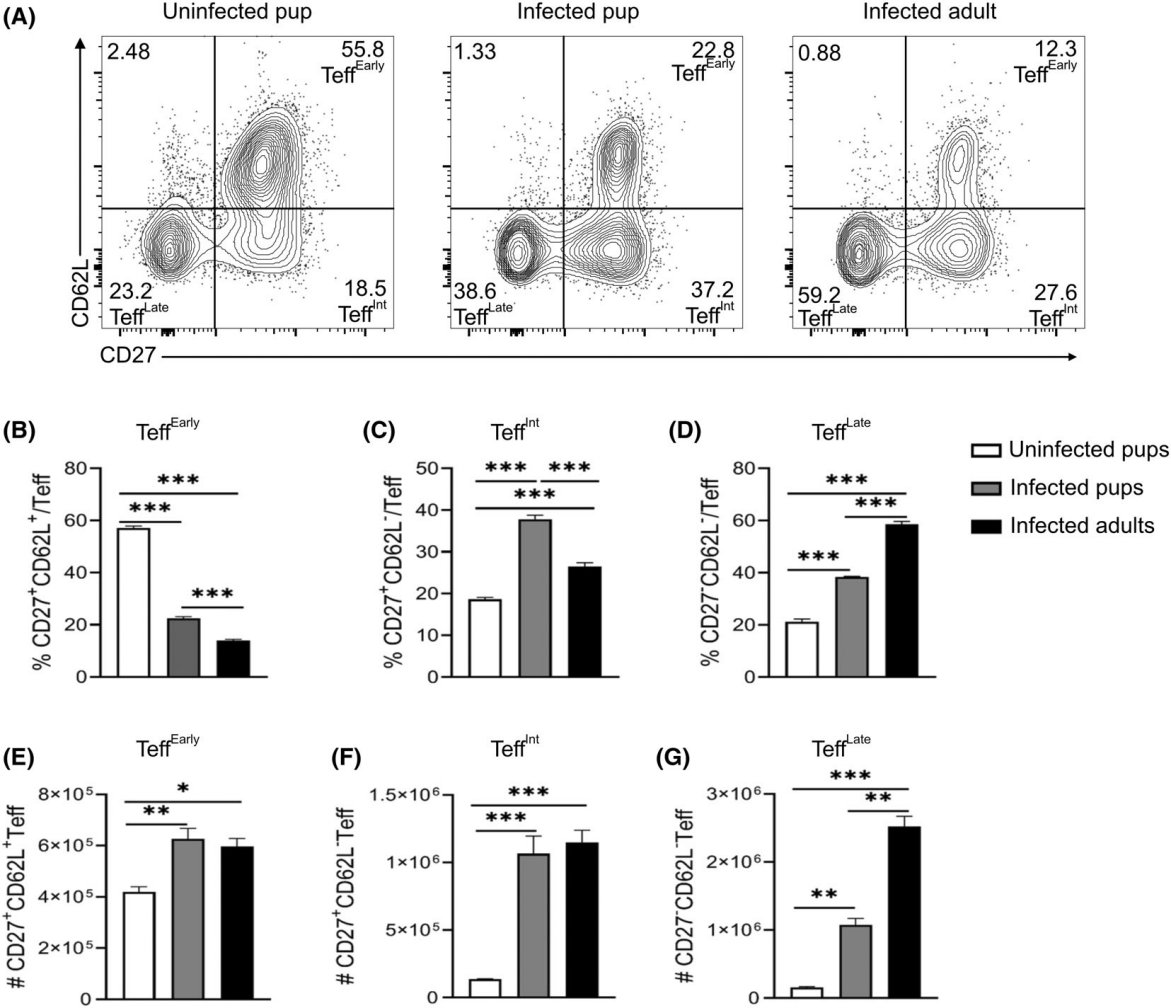
Teff subset differentiation is slower in young mice compared to adult mice after *P. chabaudi* infection. Pups and adult C57BL/6J mice were infected with *P. chabaudi* (10^5^ iRBCs), and splenocytes were analyzed at day 14 post‐infection. (A) Plots show representative Teff subsets in each group including uninfected pups (left), infected pups (middle), and infected adult mice (right). Graphs below plots show percentages of (A, B, C) or number (D, E, F) of Teff^Early^ (CD62L^+^CD27^+^), Teff^Intermediate^ (CD62L^−^CD27^+^), and Teff^Late^ (CD62L^−^CD27^−^), respectively, gated on effector T cells (CD127^−^CD44^+^CD4^+^). Data represent two independent experiments with 3–4 mice per group. The error bars represent standard error of the mean (SEM) analyzed using One‐Way ANOVA followed by Tukey's post‐test. *p < 0.05, **p < 0.01, ***p < 0.001.

**FIGURE 5:**
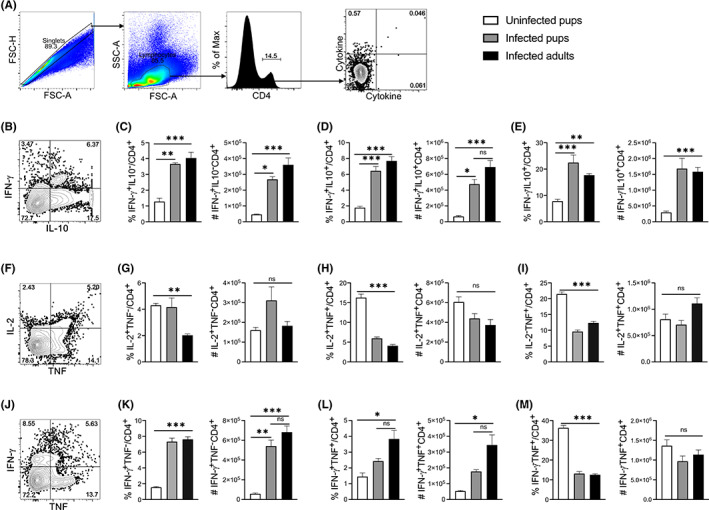
Pup CD4^+^ T cells secrete less IFNγ/TNF and IFNγ/IL‐10 double positive Th1 cytokines in response to *P. chabaudi*. Pups and adult C57BL/6J mice were infected with *P. chabaudi* (10^5^ iRBCs), and splenocytes were analyzed at day 14 post‐infection. (A) Plots show gating strategy for singlets, lymphocytes, CD4, and cytokine determination. (B) IFNγ/IL‐10 plot with quantifications of percentages and number of (C) IFNγ single producers, (D) IFNγ/IL‐10 double producers, and (E) IL‐10 single producers. (F) Plot showing TNF/IL‐2 cytokine production with quantifications of percent and numbers of (G) IL‐2 single producers, (H) IL‐2/TNF double producers and (I) TNF single producers. (J) Plot indicating IFNγ/TNF cytokine pattern, with quantifications of percent and numbers of (K) IFNγ single producers, (L) IFNγ/TNF double producer and (M) TNF single producers. Data represent two independent experiments with 3–4 mice per group. The error bars represent standard error of the mean (SEM) and analyzed using One‐Way Anova followed by Tukey's post‐test. *p < 0.05, **p < 0.01, ***p < 0.001, ns = no significance

We previously showed a linear differentiation of effector cells into three stages based on CD27 and CD62L expression.^26^ With lower Teff populations in the pups in Figure 3 above, we wondered if pup cells had any defects in the progression of effector T cell differentiation. While we observed high proportions of Teff^Early^ and Teff^Intermediate^ in the pup cells (Figure 4A–C), the numbers were not significantly different between the infected groups (Figure 4E,F). The infected adult mice had significantly higher percentages and number of Teff^Late^ (Figure 4D,G). To measure functional differences in the effector CD4^+^ T cell activation, we tested proliferation‐inducing (IL‐2) and protective (IL‐10, IFNγ, and TNF) cytokine production during *Plasmodium* infection.^27^, ^28^, ^29^ Using intracellular cytokine staining and gating strategy as shown in (Figure 5A), we observed a distinct CD4^+^ T population of double IFNγ/IL‐10 producers that are well‐defined to be protective from severe immunopathology.^12^ While the IFNγ^+^ and IFNγ/IL‐10 double producers were lower in the infected pups compared to infected adults, they did not reach significance difference in both proportions and number (Figure 5B–D). Interestingly, the IL‐10 single producers were similar in both infected groups of pups and adults (Figure 5E).

When we determined the proliferation cytokine IL‐2 together with TNFα production, we observed that apart from IL‐2 alone, which was higher in both the infected and uninfected pups, the uninfected pups have slightly higher proportions of either TNFα or both cytokines combined than the infected groups, but the cell numbers were not different (Figure 5F–I). While we observed trends toward lower proportions and numbers of double IFNγ/TNF producing CD4^+^ Th1 cells in the infected pups, this did not reach significant differences. There was also no different in single producers between the infected groups (Figure 5J–M). Taken together, these data suggest lower production of protective double cytokines may hamper the functionality of pup effector CD4^+^ T cells during a malaria infection.

### 3.3 Pup CD4 ^+^ T cells express high levels of T‐bet after *Plasmodium chabaudi* infection

**FIGURE 6:**
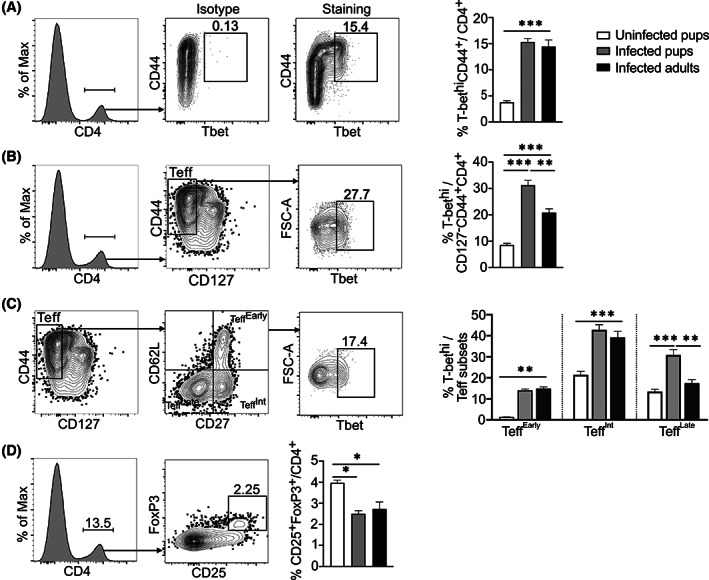
Pup CD4^+^ Teff T cells express more T‐bet after *P. chabaudi* infection. Pups and adult C57BL/6J mice were infected with *P. chabaudi* (10^5^ iRBCs), and splenocytes were analyzed at day 14 post‐infection. Flow contour plots show representative gating strategy of CD4, contour plots of (A) CD4, isotype control (middle left and sample (middle right of T‐bet expression in CD44+ activated CD4 T cells, with quantification of all groups in the right graph. (B) T‐bet expression in Teff population with plots showing the gating strategy and quantified in the right graph. (C) T‐bet expression in CD4^+^ Teff^Early^ subset, Teff^Int^ subset and Teff^Late^ subset with contour plots showing the gating strategy and quantification in the right graph. (D) Data representing gating strategy for Foxp3 expression and quantification in all the three groups. Data represent two independent experiments with 3–4 mice per group. The error bars represent standard error of the mean (SEM) analyzed using One‐Way ANOVA followed by Tukey's post‐test. *p < 0.05, **p < 0.01, ***p < 0.001

Both T helper 1 (Th1) generated by the transcription factors T box expressed in T cells (T‐bet) and forkhead box P3 (FoxP3) regulatory T cells (Treg) are important regulators of T cell differentiation during blood‐stage *P. chabaudi* infections.^30^ These transcription factors correlate with the balance in the quality of immune responses toward eliminating the parasite (T‐bet) or regulating the immune response to reduce pathology (FoxP3). Using CD44 alone to determine T‐bet in the activated CD4^+^ T cells, we observed no difference in T‐bet expression between the infected pups and adults (Figure 6A). Upon introduction of CD127 to determine T‐bet expression in effector cells (CD44^+^CD127^−^), pup effector CD4^+^ T cells expressed significantly higher levels of T‐bet after infection (Figure 6B). This increase was found to be primarily due to extended high levels of T‐bet in terminally differentiated Teff^Late^, while the Teff^Early^ and Teff^int^ were similar in both pups and adult mice (Figure 6C). Similar to previous reports,^10^ we observed a reduction in Foxp3^+^ CD4 T cells in both the infected pup and adult mice (Figure 6D). Taken together with previous studies, these results suggest a unique differentiation state of Th in pups infected with *P. chabaudi*. Despite the reduced number and less terminal differentiation state of CD4^+^ Teff in infected pups, the Teff appear to sustain expression of T‐bet into the terminal differentiated subset of Teff. If this represents a strongly polarized Th1 state, it suggests a possible mechanism for the increased behavioral symptoms seen in pups.

## 4 DISCUSSION

Malaria is a significant threat to children in endemic areas. To our knowledge, there is no good established young rodent model for malaria, thus limiting our understanding of this disease in the most vulnerable population. Our current study sought to establish a young rodent model that can be used to aid in the understanding of the immune response to, and pathogenesis of, malaria. While most of the mechanistic knowledge on *Plasmodium* infection to date relies on animal studies,^31^, ^32^, ^33^, ^34^ most of these studies use adult mice. Using our neonatal model, we showed that all infected pups had a reduced growth rate during the first 2 weeks of infection. In the 60% of young mice destined to die, the infection severely reduced weight gain over the first 3 weeks. The slower growth of infected pups observed in this study is consistent with a phenomenon seen in human studies, as malaria infection has been reported to increase the risk of stunting in children in many regions including Ethiopia and the Lao People's Democratic Republic,^20^, ^35^, ^36^, ^37^ even though these children catch up in growth with their age‐mates later in adulthood, just as observed in our experiments for pups that recovered (Figure 1B).

Similar to children who suffer more incidence of severe malaria,^38^ we observed that more pups than adult mice experience severe disease, as demonstrated by increased mortality. We also tested several behavioral assays designed to indicate sickness behavior, such as that induced by systemic cytokines in response to transient LPS exposure, and specific behavioral indicators of cerebral involvement in (Figure 2). Pups that succumbed to infection had reduced scores on spontaneous activities as well as more complex behaviors such as negative geotaxis, compared to infected adult mice or pups that survived the infection. Negative geotaxis and tremor are reflective of specific changes seen in cerebral malaria in WT mice infected with *P. berghei* ANKA, or IL‐10 KO infected with *P. chabaudi*, and are not seen in adult WT mice.^19^, ^24^, ^25^ These syndromes include extensive cerebral gliosis and vascular events including hemorrhage, edema, and endothelial congestion and coagulation throughout the brain, indicating neuropathology.^25^ Additionally, negative geotaxis, or ataxia, and reflex reaction are specifically associated with cerebral malaria and can distinguish the neuro‐specific aspects of *P. berghei* infection from sickness behavior. We anticipate to test the correlation of these behavioral changes with inflammation and leakage of the blood brain barrier in future studies.

Understanding the functionality of pup cells can provide important information for vaccine design to malaria in children, the most affected group. Thus, we investigated T cell proliferation, activation, differentiation, and cytokine production, which are key functional readouts for protective T cells. Activated CD4^+^ effector T cells were fewer in 14‐day old pups, and less terminally differentiated than adult effector T cells on day 8 and 14 of infection. Importantly, the high proportions of T‐bet in pup Teff cells indicate that they require expression of more T‐bet to differentiate into functional Th1 cells. Pup CD4^+^ T cells produced significantly less IFNγ/TNF and slightly less IFNγ/IL‐10 double positive cytokines that were not significantly different. Production of less double protective cytokines may be a contributing factor to high parasitaemia seen in the pups. Both may lead to severe malaria as seen in children. The similar levels of single cytokines in both infected groups indicate that pup CD4^+^ T cells respond same as adult mice to infection, but severity of disease may be associated with lower production of double protective cytokines.

Because single Th1 cytokines production including IFNγ and TNF, were largely similar in both pups and adults, high T‐bet levels in the pups may indicate a more highly committed Th1 phenotype. Animals deficient in IL‐12Rβ2, which is generally required for full Th1 commitment and high levels of T‐bet, are less susceptible to cerebral malaria from *P. berghei ANKA*,^39^ as are T‐bet deficient mice. But T‐bet does not appear to be required for control of parasitemia.^40^ This increase in T‐bet is strikingly different to the changes in Th1 response in neonatal mice to *Listeria*, an infection that causes strong Th1 commitment, but where T‐bet and IFNγ were down in pup splenocytes by real time PCR.^41^

While further work needs to be done to establish the role of CD4^+^ and CD8^+^ T cells in the brain of infected pups, as they are required for experimental cerebral malaria;^42^ both cell types also make IFNγ that is essential to experimental cerebral malaria in adult mice. T‐bet and IL‐12Rβ2, a marker of Th1 commitment are required for experimental cerebral malaria.^42^, ^43^ We speculate that the less differentiated pup Teff phenotype in our study, suggest an interesting pup T cell bias toward multipotency, another interesting finding to follow‐up using this new model.

Higher numbers of regulatory T cells (Tregs) are reported in children from high malaria transmission areas.^44^ Tregs require transforming growth factor beta (TGFβ) for their development and maintenance, and its presence inhibit inflammatory cytokines important for parasite elimination.^45^ This could be one reason why children experience severe disease and poor immune response, resulting in death from malaria infection. In a recent study, it was shown that Treg cell numbers decline over time in children who are heavily exposed to several malaria infections, which correlates with reduced symptoms.^10^ Similarly, we observed a decrease in Foxp3^+^ cells in both infected pups and adult mice in our current study supporting an increase in the Th1 to Treg ratio.^13^

One of the most important questions in malaria research is if age is the primary factor influencing susceptibility to cerebral malaria, or if the young age of patients is due to their exposure from birth. In unpublished data using an adoptive transfer model of Merozoite Surface Protein‐1 (MSP‐1 B5) TCR transgenic (Thy1.2) cells from both adult and pups into adult Thy1.1, we observed lower recovery of pup host cells, suggesting a differential T‐cell responses to *P. chabaudi* in pups and adults. This newly developed model of severe malaria in young animals will be important to dissect the role of variables such as maternal immunity, pregnancy malaria, or protective components of milk. Future studies in our lab will investigate if the frequency of cerebral malaria symptoms noted here and if parasitemia could be associated with cerebral pathology in young animals. In addition, we will explore the differences in B cell and antigen specific antibodies generated under the influence of T follicular helper cells (Tfh), which are also critical for malaria protection and disease pathology.^46^, ^47^ Such investigations are likely to help in identification of variables that regulate protection and pathology in young animals. Therefore, this model will help to expand our knowledge of *Plasmodium* infection and pathology, allowing application of science to reduce death rates in vulnerable young populations.

In conclusion, our data suggest that in response to *Plasmodium* infection, there are fewer effector CD4^+^ T cells generated in pups, which have a less terminally differentiated phenotype by day 8 p.i. However, the unusually prolonged T‐bet expression seen by day 14 p.i. suggests a unique Teff differentiation state in pups. Thus, our findings provide a good young rodent model to help in understanding the pathogenesis of malaria in a population that is most affected with the disease as 80% of deaths reported for malaria occur in children.^1^ Such knowledge on malaria may be beneficial to other chronic parasitic infections like *Leishmania*, tuberculosis among others. This could improve vaccine design targeting protective cells in different diseases thus reduce neonatal mortality.

## AUTHOR CONTRIBUTIONS

Conceptualization of the experiment: MMO, Experimental Design: MMO, MRS, KG, LC, CJ, RO; Data Analysis: MMO, MRS, KG, LC, CJ; Writing: MMO, MRS, KG; Editing: MMO, MRS, KG.

## FUNDING INFORMATION

This work was funded by start‐up funds from the Department of Biology at Appalachian State University to MMO, Office of Student Research at Appalachian State University to MRS and the James W. McLaughlin Fellowship Fund at UTMB to KG.

### PEER REVIEW

The peer review history for this article is available at https://publons.com/publon/10.1111/pim.12952.

## DISCLOSURE

None.

## Data Availability

The data that supports the findings of this study are available in the supplementary material of this article.
